# School Memories about Homophobic Bullying: A Study Based on Cultural-Historical Psychology

**DOI:** 10.3390/ijerph20196810

**Published:** 2023-09-23

**Authors:** Rômulo Lopes da Silva, Vera Lucia Trevisan de Souza

**Affiliations:** 1 Postgraduate Program in Psychology and Society, São Paulo State University (Unesp), Campus Assis, Assis 19806-900, Brazil; romulo-lopes.silva@unesp.br; 2 Postgraduate Program in Psychology, Pontifical Catholic University of Campinas (PUC-Campinas), Campinas 13060-904, Brazil

**Keywords:** homophobic bullying, youth, cultural-historical psychology

## Abstract

The expression of homophobic violence in schools reveals the urgency of an analytical approach to debate the impact of this phenomenon on students’ mental health. This article seeks to debate and better comprehend school memories from young gays, lesbians, and bisexuals, as well as to discuss how homophobic bullying affected their school trajectories. This study is based on cultural-historical psychology in intersection with gender and sexuality studies. In-depth online interviews were conducted with three young subjects who identified themselves as non-heterosexual. The interviews were recorded, transcribed, and analyzed through the analytical discourse tool defined as Nuclei of Meanings. The results were organized in two topics of discussion: (a) the problems associated with non-heterosexual identity in schools; (b) the search for other ways of experiencing sexual identity in school. Throughout the article, reflections were held about the challenges participants had to deal with in order to regularly attend school and be educated, as well as the obstacles they faced in building their own ways of recognizing their sexual identity. The unique ways in which these young subjects took a stand in the face of homophobic situations show new methods to create educational interventions in order to include sexual diversity and openness to different possibilities of being and acting.

## 1. Introduction

Starting school attendance is a significant mark in children’s life. In addition to children—and adolescents—spending many years attending school, it is where they meet others, and develop relationships and affection, which are intertwined with the task of learning the knowledge that has been historically created about who we are and the world we live in. Through this interweaving of events, subjects build the images that are memorized, such as the knowledge acquired, childhood friends, the teacher that taught them to write, and other relationships that can be remembered via their imagination. These memories recapture what they experienced, heard, felt, silenced and forgot, bringing back private, unique, and collective backgrounds [1].

It is through one’s memory that we can identify conflicts, negotiations, ambivalences, and resistances regarding the values, rules, and beliefs that make up the relationships established in school [2]. This is the dynamic movement of human development, in which subjects do not passively interact in relationships but negotiate the meanings of their social environment, which are experienced as a form of building new ways of existing, thinking, and acting in the world. Therefore, we can say that different social positions compete in this tense and dramatic encounter with others, and this encounter may or may not contribute to bringing new meaning to themselves and the world around them [2]. From this perspective, it is up to the school institution, school agents, educational policies, and the school system to promote multiple and diverse ways of building students’ negotiations and meanings, in order to further their development [2]. However, violence has been a historical phenomenon that pervades students’ learning processes, with a significant impact on children and teenagers’ school experiences, challenging hegemonic norms and social values [3,4].

In this article, we seek to debate and better comprehend the school memories of young Brazilians that dissented from heteronormativity, and to specifically consider how homophobic bullying affected their school trajectories. This problem has been a great challenge for education, given the increase in conservative ideas, which reveals the urgency of analytical investigations in order to understand the effects of homophobic violence in school during the process of subjective constitution and young LGBT+ people’s mental health.

In order to situate the discussion in the field, we introduce general aspects of the sexual and gender diversity debate in education, as well as the basic concepts to comprehend the experiences of LGBT+ people. We also address the idea of human development in cultural-historical psychology, emphasizing the role of language and affection in the constitution of experiences and memories. Furthermore, we introduce the research and thoughts on the evoked narratives and dialogical encounters, which helped us to understand the evidence on how homophobic bullying has impacted the experience of sexual identity in school.

### 1.1. Sexual Diversity and Homophobia in School

Education is established as a universal human right and is further guaranteed as a social right in Brazil by the Federal Constitution of 1988 as well as the National Education Guidelines and Framework Law (Lei de Diretrizes e Bases da Educação Nacional) [5,6]. The State has the dominant role of ensuring a public, free, and quality education for all, based on social values that profess citizenship, social inclusion, diversity and social transformation [6]. However, the historical development of schooling has turned it into a place that is disciplinarian, commonly normalizing and reproducing inequalities, generating challenges for those who express behaviors that diverge from heteronormative standards [4]. What prevails in the educational system is the tension and debate over gender and sexuality, which affects the social, symbolic, political, economic, and institutional disputes related to sexual diversity.

Research conducted in Latin American countries, including Brazil, reveal that LGBT+ young people face hostility in the school environment, which leads to insecurity and low representation of gender and sexual diversity in the curriculum [3]. The situation worsens when we analyze the most recent data, which reveals that sexual orientation is one of the main reasons for bullying of Brazilian students [7]. Thus, in this educational context, it is understood that young LGBT+ students are the most affected group regarding access to education in Latin American and Caribbean countries. The predominance of homophobic bullying, a particular way of showing homophobic violence in school context, deeply affects the school trajectories of LGBT+ students, as well as their interpersonal connections, often leading to them lagging in academic performance and dropping out of school [3,7].

Studies on homophobic bullying show this complex social process, which involves different actors who appear as active accomplices, passive spectators, and indifferent observers of violence [8,9]. Researchers are guided by different theoretical and methodological perspectives; these contribute to future practices that mobilize teachers, directors, and school psychologists against bullying [8,9]. Considering the objectives and limitations of this study, we point out the main challenges for the discussion of homophobic bullying, especially in a Brazilian context.

This reality constitutes the images of memories, which are part of the school experiences of non-heterosexual young people, showing that each subject had unique and singular involvements with the school. However, this is not a particular problem for these students, but for all the relational dynamics within the educational process. In Brazil, topics related to diversity and inclusion are considered a “conflicted terrain” [10] (p. 5). In the educational field, there are disputes between the political projects that seek to overcome inequalities and discrimination against the LGBT+ community, and the conservative projects that weaken and delegitimize the actions and debates on sexual and gender diversity in school institutions [10,11].

Considering all this, it is understood that education is marked by the maintenance of a homophobic social system, in which relations of power establish a type of violence that articulates heterosexism, sexual prejudice, heteronormativity, sexism, and male dominance [12,13]. This is a complex system that defines which subjects have the right to see themselves as intelligible; namely, those who meet the societally expected coherence between biological sex, gender, and sexual desire. In short, this encompasses those who have their feelings, thoughts, and desires acknowledged, accepted, and validated in social relationships [14,15]. The debate proposed by Judith Butler illuminates the understanding that gender and sexuality are regulated by historical rules that produce discourses or narratives about oneself and others, based on compulsory heterosexuality and homophobic violence [14].

Therefore, language is something to be highlighted, since it is through language that the meanings of social reality are shared and set up by subjects during interpersonal interactions. Homophobic violence is located in this complex field of social relations, and not only refers to individual attitudes, but also to the expression of psychological, social, and cultural processes that are part of the homophobic system [12]. There is no denying that homophobia, and specifically homophobic bullying, when inserted in social and discursive practices, affects the emotions, thoughts, memories, and actions of young people who are victimized by homophobic violence and bullies.

In this study, we emphasize the urgency of addressing the social and subjective impacts of homophobic bullying on the developmental processes of young LGBT+ people. These challenges exist inside and outside school, which means that we need to understand the dynamics of school relations, through which not only is homophobia allowed, but also the vast debate on diversity in education, which results in advances and setbacks in the implementation of educational policies for fighting homophobia [4,13,15]. Thus, we highlight the importance of considering an educational project from a human development perspective that values and considers social inclusion, diversity, and differences as part of the teaching–learning process and students’ subjective formation. The real situations experienced by these young people push the need to analyze their development conditions, especially the unique ways that they oppose and question the heterosexual norm as a part of their interactions inside and outside school. In this context, we use the notion of human development proposed by cultural-historical psychology as a way of understanding the constitution of young people’s subjectivity. 

### 1.2. Language and Affection in the Constitution of Memories of Young LGBT+ People

The theory of human development proposed by cultural-historical psychology, the main representative of which is Lev Semionovitch Vygotsky, understands the external environment experienced by the subject, that is, the physical, social, and cultural environment, as central in the constitution of the human psyche [16]. When deepening our understanding of this theoretical and scientific construction from the perspective of cultural-historical psychology, it is possible to notice that it does not deal directly with the theme of sexuality but offers conceptual tools to properly understand the human experience. In an integrative literature review that aimed to identify studies based on cultural-historical psychology discussing the experiences of LGBT+ people, the recent approaches of scholars who departed from this theoretical contribution to the understanding of sexual diversity were noted [17].

These studies date from the last decade and present significant analyses of social and cultural contexts as constitutive of the experience of sexual identity. However, there are still challenges regarding the use of this theory, which is often centered on the challenges posed in the teaching and learning processes, without considering sexual diversity. However, it is a fruitful field of discussion that considers the social and cultural aspects of sexual experience [17].

Based on this perspective, each person’s development involves the complex and constant task of recreating the external environment (the world of social relations that come with cultural and social aspects) in the internal environment, which is a subjective area that is constituted as a complex inter-functional system of psychological functions. Throughout this process, language works as a mediator by default, since only through words is it possible to create and internalize signs and meanings of elements that are part of what was experienced by the subjects [16,18,19].

Therefore, the possibilities for the subject to develop and become human are in their interactions and social practices, where the meanings shared between people become a way of understanding those meanings for the person that had an experience, which means that each person has to “live the meaning” of situations that happen in the environment [19]. The school environment and the schooled knowledge are highlighted in this development, since access to all of the concepts that are shared in school can amplify the meanings of the world. In this process, the dynamics of psychological functions are affected, especially language, thought, emotion, feelings, imagination, and memory, because they rearticulate and change the way the subject understands the world and themselves [1,20,21].

Thus, conditions can be offered, through school education, that allow young people to become able to access and internalize culture, as well as to build new foundations to face situations in society. In cultural-historical psychology, the development of the psyche intertwines intellectual and affective aspects, since each person is emotionally related to social reality. Affection involves emotions, feelings, and human passion, which are built historically; therefore, it is not static nor reduced to a biological aspect. While they regulate the subject’s psyche, social relations also lead to the creation of new ways of experiencing affection uniquely, allowing new possibilities of experiencing emotions, guided by human needs and reasons [21].

As we have been discussing, the images of school institutions, school relationships, and homophobic bullying are part of the social environment and the meanings given to sexuality. The way LGBT+ people participate in school leads to unique forms of appropriation and internalization, which means to turn others’ values, norms, beliefs, and historical and cultural rules into their own. In this scope, all development happens through the dramatic encounters of each subject, with the tensions, conflicts, and contradictions of social relations. This is the drama of development, in which each young person experiences reality in a unique way, guided by a specific combination of needs, reasons, and meanings.

Fernando González Rey, interlocutor of cultural-historical psychology studies, developed the concept of subjective meaning, which refers to the relationship between the symbolic and the emotional. This relationship is created by the complex social events experienced by the subjects. González Rey emphasizes that all human needs and reasons, including sexuality, go through a complex process of articulating meanings and emotions. About human sexuality, the author says:

“The sexual need, for example, becomes a reason in the process of integrating other subjective senses that come from other spheres of psychic life, which become part of the sexual reason, such as the moral, gender constitution, the quality of different emotional relationships throughout people’s lives, the sense and meaning of their bodies, etc. All these dimensions are organized within the sexual reason and characterize the type of emotions associated to it”.

(p. 145) [22]

Therefore, the subject’s psychic constitution, sexuality, and sexual identity, embrace the complex dynamics of interacting with the environment. In this context, young LGBT+ people see their emotions, thoughts, and actions invalidated by the homophobic system, through its different ways of expression, such as the homophobic bullying present in schools in the form of jokes, nicknames, prejudiced treatment, offenses, embarrassment, threats, and physical or verbal aggression [4,7]. These situations remain frequently silenced, considered to be only LGBT+ young people’s “problems” with no understanding of the social relations’ complexity that affects how they feel and experience school reality.

In this sense, memory preserves the images of how each subject connected emotionally with reality, using their emotions, feelings, reasons, and needs, which emerge and develop in their relationships with others. Vygotsky draws attention to the role of memory in creative activity, which is accomplished through imagination and other psychological functions. By evoking situations and memorized meanings, the subject is not limited to reproducing experienced sensations, because by narrating and imagining memories, they recreate reality and build other combinations of these memories. In cultural-historical psychology, dealing with human development requires entering a complex process that is contradictory and revolutionary.

The discussion of memories in scientific research is something challenging, considering that there may be factors, such as the temporal dimension, traumatic situations and the degree of stability of memories, which can interfere with the recall process [23]. However, based on cultural-historical psychology, it is understood that the memories evoked by the interlocutors are those that were significant for the constitution of their subjectivity, and meet social, cultural, and personal demands posed at the moment at which the memory of what was lived is required. Thus, the variability of memory shows the active positioning of the subject and their psychic functions, which do not remain stable but change based on the practices, desires, and tasks placed on the subject. It is a constant process of reconfiguration of ways of thinking, memorizing, and imagining, in favor of offering new ways for the subject to act in the world and with themselves [1,20].

The multiple and diverse phenomena that we experience can be directed in many directions and open us to what is new, which means the understanding of the affections that are part of us and humanize us. By considering this perspective of development, we access the concrete situations that are experienced by young LGBT+ people and their unique ways of giving meaning to their experiences, as well as the challenges in consolidating interventive practices in their education that consider sexual diversity as part of the process of humanizing society.

This article presents the results of research carried out in Brazil that tried to understand the experiences of sexual diversity based on cultural-historical psychology. Through the analysis of the collected data, we access the dynamics of social relations in young people’s experiences in different social institutions. The school institution was highlighted in their narratives and memories, which were especially marked by experiences of homophobic bullying. In this way, we ask ourselves: How did these memories of homophobic bullying affect their perception of themselves? How did social and cultural issues affect the constitution of these young people’s sexual identity? In this sense, we aim to discuss and understand how the memories of homophobic bullying of young gays, lesbians, and bisexuals affected their school trajectories. By using cultural-historical psychology and the contributions of gender and sexuality studies, we distance ourselves from individualizing and blaming views, guided by the social and historical complexity that constitute the violence experienced in schools.

## 2. Method

This is qualitative research with an exploratory approach that deems cultural-historical psychology to be a theoretical–methodological contribution, especially the contributions of Vygotsky and other scholars of the theory [16]. From this perspective, we understand the dynamics of social relations as a part of the meanings that are produced about reality. Therefore, the phenomena we investigate are accessed by approaching the subject’s experiences, and through the process of appropriating social reality [18,19].

Since we are interested in the constitution of subjects amid social relations’ dramas, we utilized dialogism, as an onto-epistemological foundation, in order to build spaces to share meanings between researchers and the subjects that participated in the study. In dialogical encounters, both take active social positions, directed to understand historical, social, and subjective complexities that made up the experiences [16,19].

Furthermore, this is considered to be interventional research since it is a way of building scientific knowledge connected to the transformation of the investigated context. This way, the intervention was intended to collaboratively build intersubjective relations, from which the meanings on experiences could be shared, through listening and dialogue, promoting tools that transform objective and subjective conditions [14]. This intervention research was duly submitted to and approved by the Ethics Committee in Research with Human Beings of the Pontifical Catholic University of Campinas, São Paulo, Brazil, according to opinion number 4,636,576.

### 2.1. Participants and Procedures

The participants of this research were chosen through the snowball sampling method, which starts with one participant nominating new subjects to be part of the research [24]. The following criteria were used: young people from 18 to 29 years old; self-identified as gay, lesbian, or bisexual; living in cities in the southeast region of Brazil. The research involved 8 participants, and for this article we considered the narratives of 3 of them, named as Lio, Giges, and João. The choice of these three participants was due to the interest and objective of this article, which is to discuss and understand the experiences of homophobic bullying in the school memories of young people. In the narratives of Lio, Giges, and João, we find reports and meanings that favored the deepening of the discussion that we propose in this article.

At the time the research was carried out, the participants were aged as follows: Lio, 26 years old; Gyges, 29 years old; John, 29 years old. According to the Youth Statute of Brazil, they are classified as in the period of youth. The three young participants attended Brazilian public schools throughout their basic education. Considering the age of the participants at the time the interviews were carried out, they retrieved school memories lived 9 and 12 years before, between the period of 2000 and 2012. 

In order to build dialogue with these participants, we conducted profound interviews guided by the following issue: “how do you believe your sexual orientation affected your social relations?”. The interviews were conducted individually through an online platform due to the sanitary conditions at the time of this research, on account of the COVID-19 pandemic. The time and place of the interview were previously set up with each participant, ensuring their privacy. Afterwards, the interviews were transcribed and organized for analysis.

### 2.2. Analytical Approach

The analysis was based on the proposal named as the Nuclei of Meaning, based on the theory from which this study is derived [25]. This analytical proposal focuses on recurrent readings of the transcripts in search of expressions with meanings regarding the investigated theme, enabling the comprehension of contexts, relationships, and recurring themes.

Participating in this process is a movement to unveil facts, phenomena, and contradictions, and to be attentive to the way in which intersubjective relations were established. The context of the COVID-19 pandemic, namely social isolation and staying at home, demanded a specific way of approaching experiences related to sexual diversity. Thus, in the dialogical nature of the meetings, it became relevant for the researcher to share their identity as an LGBT+ person. In the interviews, terms, sexual slurs, and mentions of situations experienced by the LGBT+ public were used, which brought greater openness to sharing meanings about their experiences.

The young people’s narratives presented different experiences, situations, contexts, and established social relationships, which often did not follow linearity. Therefore, the identification of social contexts and meanings that were recurrent throughout the participants’ discourses was relevant in the data organization process.

School relations stood out from these reports, as well as the meanings attributed by the subjects about the experience of having a non-heterosexual orientation in school. The narrated memories led us to acknowledge the meanings that were built in school, which were organized into two points of discussion: “the problems associated with non-heterosexual identity in schools” and “the search for solutions and other ways of experiencing sexual identity in school”. In the next section, we show the results and debates regarding what was found.

## 3. Results

### 3.1. The Problems Associated with Non-Heterosexual Identity in Schools

In this first topic, we highlight the speeches of young people about the experiences and meanings of their sexual identity in school, which were deeply marked by the presence of homophobic bullying. According to their first perceptions, the meanings of their feelings, wishes, and needs were derived from the way they were perceived by colleagues and teachers, by whom they were seen as “problems”. Having this issue during their school years, especially in relationships established in school, led to unique ways for young gay, lesbian and bisexual people to recognize themselves and live their sexual identities. Lio describes how these situations were experienced:

*“At school, this was one of the moments I had the most, I mean... problems, because even before understanding what I was, and before knowing what I did and felt, I was bullied a lot, for being who I was, my own way, not knowing or understanding anything”.*

In the narratives, the “problems” were allocated to the lack of knowledge of Lio’s feelings, sexual desires, and bodily expressions. Even before identifying as a lesbian girl, the fact of looking like one was perceived as different by her classmates, which led to her being the target of homophobia. When summarizing school memories, she unquestionably returned to the social relations between positions and social roles; some took the position of vigilantes of gender and sexuality, while the young dissidents of heteronormativity were in the position of having their bodies watched incessantly [14,26]. For Giges, a young gay man, being in this position was also a problem that kept—and still keeps—him being careful with the way he expresses himself, as he describes:

*“this was a big problem for me, mostly. Before I accepted myself, it was also a complicated process, and, like, nowadays, I think that one thing I have problem with sometimes is that we have to be very careful with our gesturing, very careful with my behavior, depending on where I am”.*

These situations in which others commented on their behavior and gesturing, their way of speaking and expressing themselves, seem to be the background of what caused the “problem”. Sexual identity seemed to be under the rule of others, their classmates, who seemed to be in a position of power, as if they knew something these young people could not know [26]. The literature regarding the studies of sexual diversity places these experiences close to the idea of “the closet,” the period during which one’s non-heterosexual orientation has still not been revealed [26]. The distorted stereotypes, the interpretations of the body, and the very language they used have led these young people to a lack of knowledge about themselves, as Lio had mentioned, making it a violence that appeared “*before understanding what I was, and before knowing what I did and felt*”. This situation was also experienced by Giges:

*“At school, I mean… during my childhood, one thing that happened is that I still didn’t even know about my sexuality and I was already… I didn’t even know what was ‘faggot’ meant, a person would be called a faggot, or be called as a homo, and I heard these slurs from some people, without even knowing what it was”.*

The homophobic bullying situations experienced by Giges seem to be similar to Lio’s narrative, especially experiencing the process of having others naming what they felt and how they acted. Lio’s memories were especially marked by homophobic violence in intersection with racism and fatphobia. These experiences turned her school years into moments of hardship, as she recalls:

*“School was a very hard time, very, very, very hard, because it has always, and I mean always, since very little, when I was a little girl, I mean… I already felt that other kids were bullying me, I mean, and I understand that there were reasons not only because I was black, because I was fat, but also because of elements related to my sexuality […] as I was growing, sometimes boys would go and say: ‘oh, you’re a dyke”, like in a joking way, which was not fun”.*

The sayings that these young people recall show us the need to understand the social norms and values that circulate in schools, mainly those affected by the heteronormativity that shapes life in society and is undoubtedly imparted in school. Sexual and gender norms, in intersection with racial markers, create expectations on how to behave, and are regulated by hegemonic models of masculinity and femininity that are supposed to be guided by sexual attraction to the opposite gender. It is essential to discuss how non-heterosexual young people experience school in a unique way when they do not correspond to such expectations. The formal aspects of education often hide this issue affecting the subjective constitution of young people who spend long years attending school daily.

In their narratives, we can observe the affective nuances that are constituent parts of their sexual identity, and the appropriation of school knowledge that comes pierced by emotions and feelings that affect the dynamics of these relations, turning experiences unique, which means each one experiences their time at school in a different way. In this sense, amid the singularities experienced by young people in situations of homophobic bullying, there seems to be an emphasis on certain positions and social roles played in school relations. This seems to be taken on the realization that dissenting from heteronormativity would turn them into unintelligible people, which means unable to be understood and, therefore, into victims of violence. The debate on intelligibility and unintelligibility was conducted by Judith Butler, who defines that people become intelligible “when acquiring their gender in conformity with recognizable standards” [14] (p. 42). According to the author, the standard that is recognizable and intelligible is related to the coherence between sex/gender/desire, as defined by heteronormativity. Thus, being unintelligible means not being accepted and represented by the norm, but instead considered vile; that is, being prohibited and rejected.

Putting this debate into a school context seems emblematic, because when we consider the historical and social conditions that determine how schools are structured, acceptance and belonging seem to be a paramount condition for education. However, students’ experiences show that, in order to be accepted in school, they need to internalize the values, culture, and sexuality-related behaviors previously set so that, only then, they would be intelligible. This way, we question the role of school in the process of making LGBT+ people more intelligible or unintelligible, when genders and sexual desires outside heteronormativity are put under the sign of violence. By recalling the interviewees’ narratives, João recollects the situations when it was not possible to be recognized as intelligible, accepted, and represented, as follows:

*“I couldn’t say that, I couldn’t express these things that happened, right, these embarrassments, these moments with no respect and even some moments when I was almost injured, because I have been beaten up, too, when I was a child, as I remember from school, so, I couldn’t say it, I didn’t know how to tell anyone, and in those times we had a mindset according to which this is wrong, that you are the one who’s wrong, and you don’t know what to do”.*

João’s narrative shows that the features indicating dissident sexual identities led young people to be marginalized and excluded, not allowing them to be understood and accepted. However, this is a relational process in which they negotiate reality to promote encounters and relations that allow them to feel accepted and welcome. One of the triggered movements refers to the search for dialogue with educators about the experienced violence. Giges recalled the moments when he tried to reach adults, his teachers, to speak about and face the real problem: homophobic bullying. However, these were unsuccessful attempts:

*“Something that made me suffer was seeing that teachers noticed the prejudice against me, being bullied in school, and wouldn’t do anything to help. […] I asked for school support, you know? Because of the bullying and so, and I was almost expelled from the school. […] They never assaulted me physically, but they verbally abused me, you know? By swearing and all, and the teachers would see it and do nothing. At some point I lost control, in two moments I lost control and got really mad. Once I had a crying crisis and went to the administration office. They called my mom and I went home, I didn’t tell them what they were calling me. That they were calling me gay. I just told them they chased me, but didn’t say the reason, you know? I couldn’t talk about it. So they arranged a meeting, and it kept going. There was one time I lost control to the point of aggression. This time, when I intended to beat one of the boys, people gathered in classroom to hold me. Then I went to the administration office and they wanted me to be expelled”.*

Giges’s experience is similar to that of João, since both of them could not talk about the homophobic bullying. Language, and more specifically the mastery of speech, is key to the relationship between people, in external environments, and is converted into the relationship to oneself, in the internal environment, mediating ways of speaking and expressing the thoughts and feelings. The experiences of Lio, Giges, and João reveal that the “problem” and difficulties related to their sexual identities involved a lack of space for dialogue with others, so that these young people could take ownership of how to speak and say what they felt.

The acknowledgement of sexual identity has been pervaded by many dramatic social situations that they had to face. Within the educational environment, there were many “problems”, including the representation of adolescence and youth, which was seen primarily through the negative side, marked by a hard period with emotional instability, rebellion, and constant opposition to adults [27]. Such representation seemed to promote the naturalization of violence and the concealment of social situations that would be the basis of conflicts. This idea of naturalizing their experiences hides the social and cultural source of the “problems” in school. In Giges’s experience, in the moment he reaches for school support, there seems to be a naturalization of homophobic violence in school relations, as a by-product of the lack of spaces for the construction of dialogues about differences and diversity. The experiences they share suggests that dissident sexual identities have continued to be deemed unintelligible, as something to be excluded from school.

Through recalling school memories, Lio shared that the very few places where it was possible to talk about sexuality were dominated by religious and conservative speech, as we can see below:

*“I remember that during High School, a pastor attended school to lecture about these things, and I remember one that had a strong impact on me. He talked about relationships, about the relationship between a man and a woman, and he was… kind of encouraging virginity, really, and how important it is to wait for marriage and have sex only with your husband or wife”.*

This was one of the few moments to think and talk about sexuality, which was undertaken from a heteronormative point of view. The narratives in school pointed to Lio that “*this was a problem for me*”, which corresponds to the strategy of the homophobic system of individualizing young people and holding them accountable for the violence committed against them. The religious speech at school, which seems to perform the role of sexual education, determines what can be said, experienced, and felt in school. Facing this reality, we continue asking about the spaces for fighting against homophobic violence. As Giges and Lio did not find them, neither did João:

*“There was never an intervention, no, it didn’t exist, at least not in the schools where I’ve studied. It was common noticing that what was going on was prejudice or bullying, as we say today, and the worst that could happen was to be taken to the principal, get a referral, I ended up getting one, how do you call it? Suspension, because I hit someone for self-defense, I had to hit them so I wouldn’t be beaten up”.*

The images of school show that oral language is restricted to offenses and jokes, not having ways of recognizing differences and diversity as part of students’ identities. João’s story, as Giges’s does, shows that physical aggression became a way of dealing with homophobic bullying, but these attempts continued to have them being held accountable for the prejudice. The big problem they experienced was related to the fact that no one in school could name the reasons that led the bullies to victimize them, as well as the problem of not having anyone to talk to, and therefore not accepting themselves as LGBT+.

When they were revealed in school relations, all gay, lesbian, and bisexual identities were put into the field of the “unthinkable” and “unspeakable”, and turned these subjects into despicable bodies, for deviating from heterosexual norms [14,28]. With that in mind, what resonates as possible evidence of the impact of homophobic bullying in the formation of young LGBT+ people is the creation of a “non-place” in school, where one cannot talk about their desires and feelings that go against the social hegemonic norm. It is of extreme importance to comprehend the process of turning these young people into unintelligible from inside the school walls, because it is through accessing and owning school content that they can have a qualitative leap in their understanding of the world and themselves. In that sense, it is important to ask how they found solutions to create forms of recognizing their sexual identity, expanding the meanings shared at school.

### 3.2. In Search of Solutions and Other Ways of Experiencing Sexual Identity in School

From the perspective of cultural-historical psychology, we debate the meanings created by these young people, according to their experienced social relations and the conditions, to internalize how they deal with their own sexuality. This way, the experience of homophobic bullying is not restricted to each individual, but also brings dramas that are developed collectively. This way, in the dynamics of school relationships, we can see that the students’ experiences have been changing, going down a path that begins with an apparent lack of knowledge about their own sexuality, as well as the non-acceptance of their sexual identity, towards the search for ways of coping with situations of homophobic violence.

As moving towards good experiences was a challenge in their school relations, a new meaning was created: that strategies to fight homophobia would be found outside the school. The lack of space in school, as well as the refusal to create ways of fighting homophobic bullying, seemed to lead them to individualization, which means they had to be held accountable when attacked, and also find the affections that would help them to accept their own sexual identities. Recalling Giges’s memories, we highlight the school’s guidance for approaching his feelings, affections, and dramas:

*“I said I needed someone I could talk to in that moment, right, and then I told her [educational coordinator] what I was feeling and said I felt really bad, that I didn’t wish to live anymore and didn’t want to do anything. Then she told me:—Giges, you know, I think you have depression and so. And she said it like this:—I can’t help you, I mean, I can’t do anything for you, you would have to talk to your family and ask for help”.*

According to his narrative, the school individualized homophobic situations, centralizing in the students their reasons and complaints about the effects of homophobic bullying. This situation raises challenges for pedagogical and psychological interventions aimed at sexual diversity in the school environment, especially given the urgency of understanding how these social dynamics are part of the subjects’ experiences. By looking at the relations between the subject and the social environment, we see the possibilities for these young people to negotiate their meanings of their experiences. This way, the causes and reasons for homophobic bullying are not placed on a one-way path, determined by something from inside out, but guided by multiple meanings that are produced in the complex and dialectical encounter the subject has with their social environment.

The meanings that were shared in school about their sexual identity added barriers that stopped them from accessing affections that could grant support, acceptance, and welcome. However, we highlight that the images of narrated memories are not final, since they envision the countless possibilities that open up to find spaces where they can establish intersubjective relationships. These spaces allow for the sharing of different meanings in encounters with others that encourage building new ways of recognizing oneself and establishing social relations. These challenges have led them to imagine and find other trajectories, where sexual identity is not exclusively marked by violence and denial.

Giges recalls the moment when he found spaces outside school to build other meanings around his sexual orientation:

*“when I got to the first year in High School, I made friends with people in town who others were kind of afraid of, you know. [laugh] I started being friends with them, usually the rock music folks, you know, the time of metal heads, I started being friends with these people, because I also enjoyed the music. Then I started walking around with them, so I believe this helped me a lot, you know why? People started to be afraid of me, because I was around these friends, so this was a period when I didn’t suffer much prejudice, by fear, you know, for being with those people”.*

The “*rock music folks*”, as named by Giges, were a group in which his sexual identity was not a problem, since the way they dressed with dark clothes and thick chains, as well as the content of the music they listened to, which was based on diversity and difference, offered him the acceptance that he did not experience in school. The stereotype of “*metal heads*” became a way for Giges to avoid jokes and offenses he had to deal with in the school environment, since it created fear in his classmates. Music is shown as a possible indicator of the solution he found to face the meanings that were narrowed down to violence, with no space to talk and recognize feelings:

*“in these moments of loneliness, music was my company. So I would listen to music, see the lyrics and identify with the lyrics, sometimes many songs had something that represented that moment I was living. Usually heavy metal songs are the best for when you are facing moments like, ‘I wanna die’, you know [laugh], because the lyrics are heavy and there’s always a song about being chased, suffering prejudice, you know, feeling there is no place for you in this world, because these songs are basically all that, about that. So these were lyrics that translated what I felt”.*

Giges highlighted that, in this moment, fear was possibly what led his classmates to reduce the homophobic attacks and opened spaces for him to find other meanings. Thus, we can see art, and music more specifically, as something that motivates new meanings on affection, which can be noticed by the importance of knowing songs that guided his LGBT+ experiences, as he describes:

*“I started listening to Madonna at that time, and her songs are something I always tell people, it was songs like those that made me get rid of all the feelings that I had, you know, the scolding I had with myself, and Madonna’s songs were very important for me to accept who I was, as well as her video clips and her story. My last year in High School was a year when I listened to many songs of hers, and it was very supportive, extremely, extremely supportive, you know. I say that Madonna was one of the people who saved my life”.*

For Giges, the spaces outside school, such as the group of friends in his town, where his sexual orientation is not considered a “problem” but understood as a possibility to experience sexuality, have allowed him to experience songs, movies, and social media groups that comprehended LGBT+ experiences. Art is emphasized by manifesting, with language, a way of portraying the feelings of exclusion and not belonging that he experienced at school. João and Lio reminded us that, during their childhood and adolescence, they could see some positive images of LGBT+ identities in the media. Movies and soap operas showed kisses between people of the same gender, as well as new discourses about sexual diversity. These were also ways of building new meanings for sexuality.

However, the challenge of sharing these meanings in school remained. For João, what prevailed in his memories was the flexible position he held in school relations:

*“for a long time this was a problem for me, you know, the prejudice, and how people dealt with me, and later I realized that it wasn’t, I think after a while we end up accepting it, let’s put it that way, some jokes, we start adapting somethings, so we get more accepted”.*

For him, his whole school experience was defined by non-acceptance, but he still managed to accept his sexual identity. João recalls that, since his adolescence, he has been to psychotherapy, which meant the “*time when I started to see myself, pay attention to me, this issue of who João is, João’s sexuality, and get deeper in that matter, because I realize the more you know yourself, the more self-assured you feel about yourself, the less other people and external factors influence you*”. Therefore, psychotherapy is considered a strategy to face homophobic bullying. Regarding this, it is still necessary to bring up the actions and relations that change in the school institution, because it seems that in João’s memory images, there was a confrontation between him alone and all the others, as he claims: “*we start learning that what the others see in you is much more about themselves than about you, so the problem is not me, it’s the others*”.

By highlighting João’s experiences, we cannot forget the experiences brought up by other young people, since we have seen meanings that are shared and are part of a narrative that talks about the place that others occupy in the constitution of sexual identity experiences. Henri Wallon, an important interlocutor of cultural-historical psychology, states that “The socius, or the other, is the ego’s constant partner in mental life” [29] (p. 165), which can be seen in these young people’s narratives; namely, the presence of others in their relations with the social environment and themselves. Experiencing a gay, lesbian, or bisexual identity in school involved dealing with others, who tried to impose their meanings on the constitution of masculinity and femininity through homophobic bullying. Therefore, these young people were forced to negotiate ways of being accepted in school, as well as ways of accepting their own sexual identity.

We noticed that experiences of homophobia in school engendered the challenge of dealing with others that reproduced homophobic bullying, and often resulted in the desire to choose not to have any relationship with these others [26]. There is no denying that the “*others*” refer to the homophobic system, which is not left in the past, but remains in the present, demanding constant confrontations and negotiations. It seems that, in this encounter with others, these young people’s desires were directed towards not experiencing the silence and non-acceptance that appears in intersubjective relations; instead, it is about taking action and opposing the stereotyped, offensive, and violent images that appeared in school regarding their sexual identities. In this complex process, they had to learn different ways of speaking for themselves, which was, for João, “*a liberation, a liberation that was, once and for all, speaking for myself and not letting others do it for me*”. 

In that sense, the search for strategies inside and outside school is related to the construction of a true knowledge about their sexual identities. For Lio, it seemed much more complex due to the intersection of other social markers beyond her sexual identity; namely, being a woman, overweight, and black. She recalls that only after leaving school was she able to understand what happened in the school relations, such as homophobic bullying, racism, sexism, and fatphobia, throughout all school years. Lio confirmed she was “*living in celibacy*” and “*started to understand I’m a black woman, like, in the last term at university*”, which seems to reveal that Lio found, belatedly, spaces and social interactions to talk about experiences related to sexual and racial identities.

Lio’s memories show that homophobia in school occurred in intersection with other social markers, such as gender and race, intensifying the silencing of her singularities. The violence in school favored the obliteration of her identity, leading her to be excluded and put in a place of silence. However, she questioned this constantly, and as Giges did, she found in art, more specifically in music and literature, ways of processing what she experienced in school. Lio recalls that some songs were about her life; they therefore became a space to find herself.

Many were grateful for the ways they found in order to deal with homophobia in school, which shows the need to understand the singularities of school experience and homophobic bullying situations. In their experiences, the search for spaces to speak and listen stand out, as does art. Art became a relevant place in their experiences, transforming their feelings, emotions, and their understanding of their experiences. These dynamics of appreciating art are debated in cultural-historical psychology as a human production that can affect the subject and stimulate both the imagination and new ways of relating to reality and oneself [18,30]. Once we recognize its humanizing potential, we can see that these young people’s memories show potential strategies of intervention in school, in which sexual diversity could be approached by enjoying works of art that report and fight homophobia. In the stories of school memories, each one of them describes in their own way how art was paramount to amplify their meanings about sexuality.

When Lio, João and Giges share their memories, they recall dramatic situations that followed their developmental trajectories in the school context. Within cultural-historical theory, these situations are situated in a dynamic of tensions, conflicts, and negotiations that persisted through many years and intensified due to the non-acceptance of sexual dissidence. The images of school memories show the social role they were able to take in this dramatic event; when their own acknowledgment and acceptance of their sexual identity to themselves led to being unintelligible and victims of violence, they insisted on talking about their diverse point of view. The experience of gay, bisexual or lesbian identity is not restricted to an intimate and individual position but demands the analysis of the homophobic system that affected their relations, as well as urgent and effective interventions that transform school relations.

## 4. Conclusions

In this article, we analyzed the school memories of young people who were dissident from heteronormativity, as characterized by homophobic bullying. The tensions and conflicts that were present in their school relations arose at first as confused and partial images of their internal and social realities. These images, affecting the subjects but also being affected by them, were conceived according to the way school limited how and when sexuality should be approached. In this process, there was a constant negotiation of meanings and comprehensions, which are directly related to the social role they could fulfill in school interactions.

Acceptance and non-acceptance are present in this place of confused images and affected young people in a way that led dissident sexual identities to belong to the unspeakable and unintelligible group; yet, these subjects act in order to find true knowledge, and in search of strengthening the power of existing. Therefore, it was necessary for them to have a say, even belatedly, after the experiences of suffering in their social relations, yet some of these relations enabled the appropriation of new knowledge and ways of meaning in reality, removing the immediate concrete environment that was characterized by homophobic bullying.

This movement is close to what Vygotsky defines as a naming process, which leads to changing the function of speaking. In the author’s words, this process can be understood as following:

“Initially, speech follows action, provoked and dominated by activity. Afterwards, however, when speech is transported to the beginning of the activity, there comes a new relation between word and action. In this moment, speech will direct, determine and dominate the course of action; there comes the speech planner function, beyond the existing function of language, which is of thinking the external world […] this structure itself can be changed and reformed when children learn to use language in a way that allows them to go beyond previous experiences when planning future actions”.

[31] (pp. 37–38)

Thus, we note that, when debating the appropriation of speech in human development, Vygotsky is not restricted to childhood but refers to a process that may occur throughout development, as we observe in the experiences of the subjects we interviewed. Speech, for example, is present throughout the reconfiguration of young people’s psychological system, as well as in the social environment where they are, since they can think, plan, and act in the aim of overcoming affections that kept them effortless and powerless. According to Vygotsky, “Word was not in the beginning. In the beginning, there was action. Words constitute the end rather than the start of developing. Words are the end that crowns actions” [32] (p. 485).

With all this considered, by turning back to the memories of the interviewees, the presence of acceptance and non-acceptance occurs among historical and social contradictions that are part of school institutions. These contradictions offer meanings on sexual orientation that, at first, by silencing and denying their experiences, corroborate to reducing the power of being of these young people. However, in accordance with Vygotsky, we can reinforce that “the concept or meaning of word can evolve and the development itself is a more complex and delicate process”, [32] (p. 248); this Vygotskian theory is supported by the concept of “subject” in cultural-historical psychology as an active and creative being, who is in constant negotiation with the environment and oneself, in order to take on the social roles that enable an increase in the power of action.

In this way, the social situations restricted by silencing, non-acceptance, and homophobia and other ways of denying young people’s experiences have become part of the reasons that directed them to own their speech and create new meanings, in which they are open to new possibilities of imagining and living the acceptance of themselves. Access to language and speech, in the school process, creates possibilities for students to expand the meanings of the world and themselves, as well as to create ways of relating to the social environment. Recovering these memories allowed us to not only discover these bullying situations, but also learn about the subjects’ progress on their development process, despite obstacles and challenges. Mostly, it allowed us to consider the urgency of interventions in the school space that encourages the fight against homophobic violence.

## 5. Limitations and Future Directions

This study has an exploratory character and focuses on the school experiences and memories of young people who self-identify as gay, lesbian, or bisexual. It comprised a small number of participants, which makes it impossible to propose generalizations to the experiences of the population in question in general. However, due to the characteristics of the participants in this study, we understand the difficulties in accessing LGBT+ young people who were willing to share sensitive topics, such as homophobia and social exclusion. Even so, we understand that the theoretical–methodological objectives and foundations that guide this study support new analytical propositions that aim to deepen the understanding of the unique and particular experiences of LGBT+ young people.

The research was centered in the southeastern region of Brazil, thus presenting particular experiences in this context, especially those experienced by young people who entered and completed their schooling process. Therefore, we recommend that future research consider the relational dynamics experienced in educational contexts in recent years, in addition to having research carried out directly in schools. Given the context of the COVID-19 pandemic experienced during the research development period, it was not possible to carry out such articulation in the school context. In addition, we emphasize the importance of addressing how the situations experienced at school affect other social contexts experienced by young people, such as family, work, and higher education.

Another aspect that should be considered in future studies is the importance of deepening the discussion based on intersectional analyses that are able to cover the complexity of the intersections between racial, gender, class, sexuality, and generation, among other related social markers, articulating with the experiences of young LGBT+ people. Intersectionality, as proposed by black feminism, starts from the understanding that social markers overlap and function in a unified manner, revealing unique ways of experiencing social reality [33].

## Data Availability

Data are available on request due to restrictions e.g., privacy or ethical. The data presented in this study are available on request from the corresponding author.
